# Diffuse Alveolar Hemorrhage due to Acute Mitral Valve Regurgitation

**DOI:** 10.1155/2013/179587

**Published:** 2013-12-08

**Authors:** Creticus P. Marak, Parijat S. Joy, Pragya Gupta, Yana Bukovskaya, Achuta K. Guddati

**Affiliations:** ^1^Division of Pulmonary Medicine, Department of Medicine, Tahlequah City Hospital, Tahlequah, OK 74464, USA; ^2^Department of Internal Medicine, University of Iowa Hospital, University of Iowa, Iowa City, IA 52242, USA; ^3^Division of Pulmonary and Critical Care Medicine, Montefiore Hospital, Albert Einstein College of Medicine, Yeshiva University, New York, NY 10467, USA; ^4^Department of Pharmacy, Massachusetts General Hospital, Harvard Medical School, Harvard University, 50 Fruit Street, Boston, MA 02114, USA; ^5^Department of Internal Medicine, Massachusetts General Hospital, Harvard Medical School, Harvard University, Boston, MA 02114, USA

## Abstract

Diffuse alveolar hemorrhage (DAH) can be caused by several etiologies including vasculitis, drug exposure, anticoagulants, infections, mitral valve stenosis, and regurgitation. Chronic mitral valve regurgitation (MR) has been well documented as an etiological factor for DAH, but there have been only a few cases which have reported acute mitral valve regurgitation as an etiology of DAH. Acute mitral valve regurgitation can be a life-threatening condition and often requires urgent intervention. In rare cases, acute mitral regurgitation may result in a regurgitant jet which is directed towards the right upper pulmonary vein and may specifically cause right-sided pulmonary edema and right-sided DAH. Surgical repair of the mitral valve results in rapid resolution of DAH. Acute MR should be considered as a possible etiology in patients presenting with unilateral pulmonary edema, hemoptysis, and DAH.

## 1. Introduction

Hemoptysis can be caused by lesions that are localized in the airway, lungs or by widespread lesions in the lungs. Diffuse alveolar hemorrhage (DAH) is characterized by widespread bleeding into the alveoli due to microvascular injury [1]. DAH may also be accompanied by pulmonary edema. The resultant impediment of gas exchange is thought to cause dyspnea. Whereas most patients present with bilateral involvement, unilateral involvement is rare. Cardiogenic unilateral pulmonary edema is a rare clinical entity that presents with diagnostic challenges. Most cases occur in the upper right side and are caused by severe mitral regurgitation [2, 3]. It is associated with an independent increased risk of mortality due to possible delay in diagnosis and underestimation of the severity of mitral regurgitation (MR). Cardiogenic unilateral pulmonary edema is more common on the right side for several reasons. The direction of the mitral regurgitation jet predominantly affects the upper right pulmonary vein and causes a larger increase in mean capillary pressure on the right side. This may consequently result in a greater degree of right acute pulmonary edema.

Patients with DAH usually present with dyspnea and diffuse alveolar infiltrates noted on imaging. Many etiologies such as drug exposure (penicillamine, propylthiouracil, ketorolac, etc.), cocaine smoking, pulmonary embolism, sarcoidosis, vasculitis, and mitral stenosis have been documented to cause DAH [4–8]. However, acute mitral regurgitation (MR) has rarely been reported as an etiology for DAH [9–13]. Cardiac etiologies of DAH seem to act through mechanical pressure rather than by inflammation of the capillaries as seen in most other causes of DAH. Diffuse alveolar hemorrhage is diagnosed by sequential bronchoalveolar lavage revealing increasing hemorrhagic lavage returns.

## 2. Case Description

**Figure 1:**
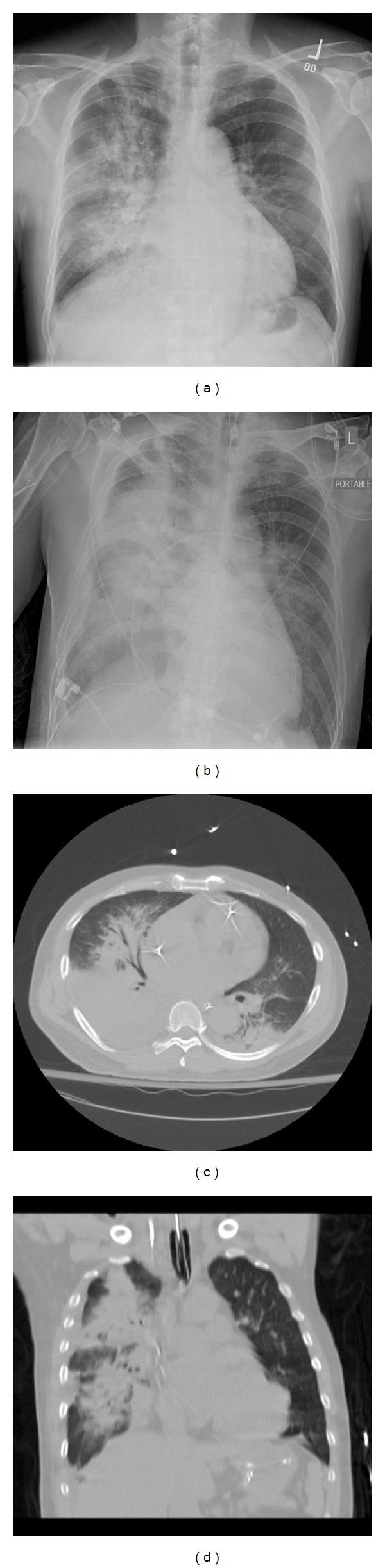
(a) CXR showing right sided fluffy infiltrates during the initial presentation. (b) CXR showing a worsening of infiltrates accompanied by pulmonary edema. (c) and (d) Sagittal and transverse chest CT sections confirming the presence of dense infiltrates predominantly located in the right upper and middle lobes.

**Figure 2:**
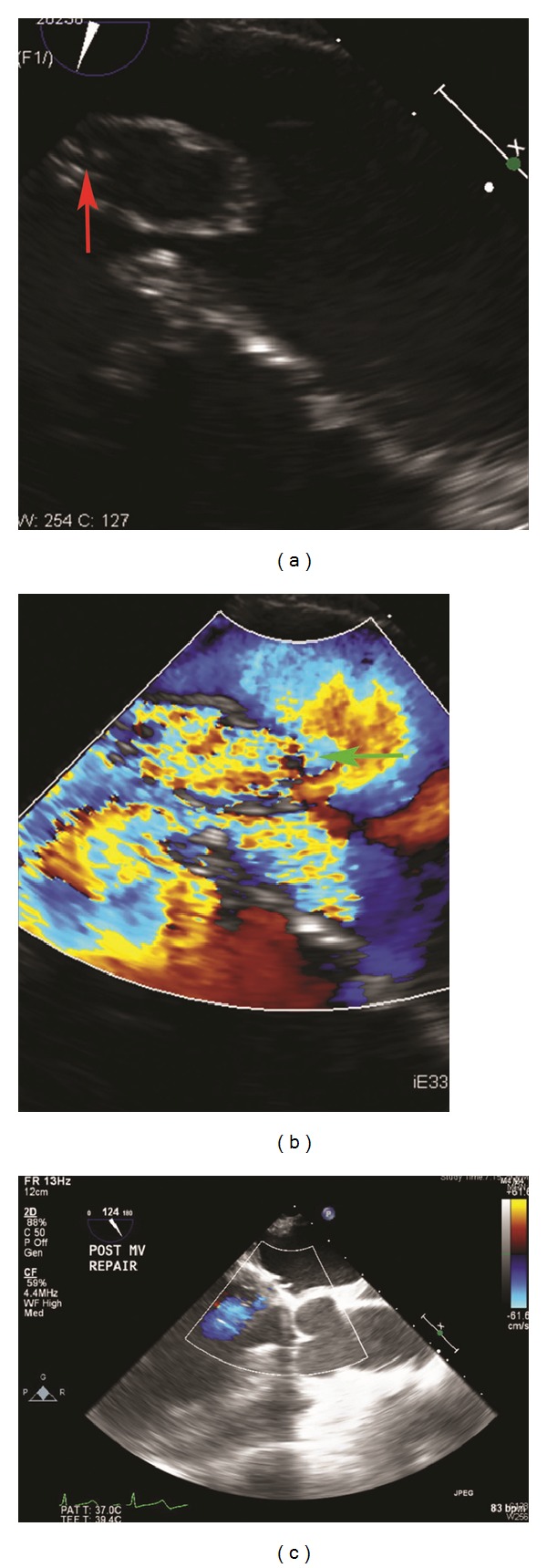
(a) and (b) Perioperative TEE revealing a perforated posterior leaflet (red arrow) and regurgitant flow directed towards the right side (green arrow). (c) Postoperative TEE showing no evidence of mitral valve regurgitation.

**Figure 3:**
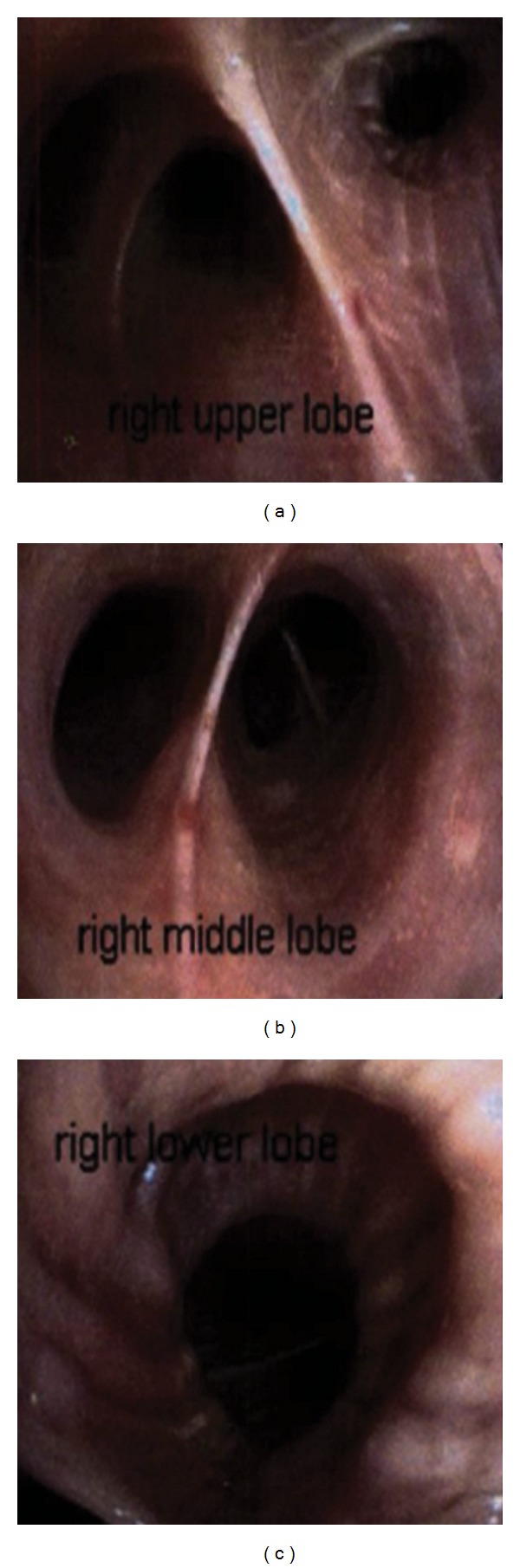
(a), (b), and (c): Repeat bronchoscopy 3 days after the mitral valve repair showing clearing of the alveolar hemorrhage in all lobes of the right lung.

**Figure 4:**
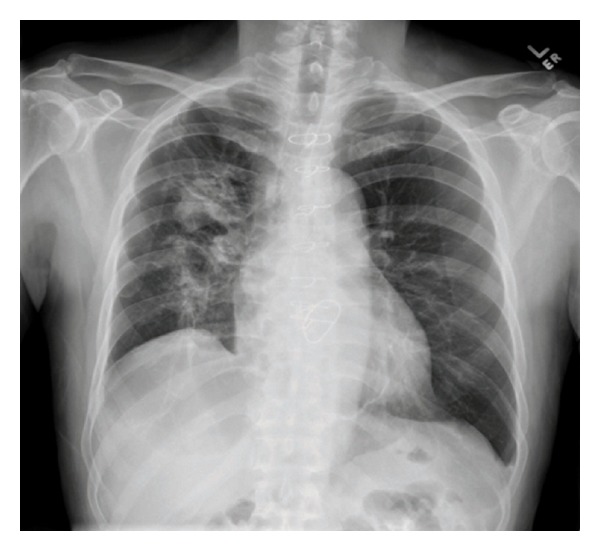
CXR after a few weeks of mitral valve repair showing clearing of the infiltrates.

A 57-year-old Chinese male with a past medical history significant for hypertension, hyperlipidemia, and moderate mitral regurgitation secondary to mitral valve prolapse presented with a 3-week history of progressive hemoptysis and worsening dyspnea. The symptoms started insidiously and progressed to a point where his exercise tolerance was reduced to a few steps. He was a plumber by profession and a lifetime nonsmoker. He denied wheezing, fever, night sweats, chest pain, and weight loss. He had no history of hemoptysis or bleeding from any other body sites. He did not use any medications and denied recent travel or sick contacts. On physical examination, he appeared to be in moderate respiratory distress, tachycardia, and tachypnea. His vital signs were blood pressure of 146/80 mmHg, heart rate of 116/min, respiratory rate of 34/min, temperature of 99.1 F, and saturating at 96% on 3 liters of oxygen by nasal cannula. He was oriented to time place and person and was found to be using his accessory muscles of respiration. His chest examination was notable for coarse crackles on the right side without any wheezing. His cardiac examination revealed tachycardia with a 3/6 pansystolic murmur best heard over the mitral area. The rest of his physical examination was unremarkable. His extremities were perfusing well and no peripheral edema was noted. His labs were notable for leukocytosis with neutrophilia (WBC: 12.7 k/mm^3^; neutrophils: 84%; eosinophils < 1%), his electrolytes were within normal range, and renal function was deranged with elevated blood urea nitrogen (BUN) and creatinine (Na: 143 mEq/L; K: 4.6 mEq/L; Cl: 107 mEq/L; HCO_3_: 23 mEq/L; BUN: 40 mg/dL; creatinine: 1.5 mg/dL). His liver function tests (LFTs) were notable for elevated liver enzymes (AST: 48 U/L; ALT: 127 U/L). His arterial blood gas (ABG) was notable for a pH of 7.44, pCO_2_ of 31 mmHg, and a pO_2_ of 84 mmHg. His chest X-ray (CXR) showed right sided fluffy infiltrates (Figure 1(a)). He was admitted to the medical floor and started on ceftriaxone and azithromycin for presumed community acquired pneumonia. However, his respiratory status deteriorated and he was transferred to the Cardiac Intensive Care Unit where he was started on noninvasive mechanical ventilation (inspiratory pressure of 15 mmHg, expiratory pressure of 8 mmHg at 100% oxygen supplementation). An ABG showed a pH of 7.13, pCO_2_ of 64 mmHg, and a pO_2_ of 68 mmHg indicative of acute respiratory acidosis. His respiratory condition continued to deteriorate and he was intubated and mechanically ventilated with a positive end expiratory pressure (PEEP) of 5 mmHg and 50% oxygen supplementation. The patient's CXR revealed infiltrates which were more dense and confluent (Figure 1(b)). His labs were significant for up-trending leukocytosis (15 k/mm^3^). The patient's blood and urine cultures were negative. Tests for legionella, mycoplasma, HIV, and influenza were also negative. Autoimmune and vasculitis panels [antinuclear antibody (ANA), antineutrophil cytoplasmic antibodies (ANCA), and antiglomerular basement membrane antibody (GBM)] tests were negative. An ABG after commencing mechanical ventilation showed a pH of 7.44, pCO_2_ of 31 mmHg, and a pO_2_ of 81 mmHg. Computed tomography (CT) of his chest confirmed the presence of dense infiltrates predominantly located in the right upper and middle lobes (Figures 1(c) and 1(d)). Transthoracic echocardiogram showed an ejection fraction (EF) of 70%, mildly dilated left atrium, significant prolapse of the posterior mitral leaflet, and moderate tricuspid regurgitation. Right heart catheterization showed a pulmonary artery pressure of 56/23/35 mmHg (systolic/diastolic/mean); pulmonary capillary wedge pressure (PCWP) of 22 mmHg, right atrial pressure of 4 mmHg, RV: 53/6 mmHg (systolic/diastolic); cardiac Index (by thermo dilution) of 2.1 liters/min/m^2^, and a pulmonary artery resistance of 9 wood units. Bronchoscopy revealed fresh blood in all the lobes with no obvious source and no endobronchial lesions. Sequential lavage from the right middle lobe was not progressively bloodier and hence less consistent with diffuse alveolar hemorrhage. Bronchoalveolar lavage was negative for cytology, acid fast bacillus (AFB), fungal stain, and pneumocystis carinii pneumonia (PCP). Thoracentesis yielded 150 cc of serous fluid with a pH of 7.6, LDH of 80 U/L, protein of 0.9 g/dL, glucose of 120 mg/dL, cell count with a differential of 55% of neutrophils, lymphocytes of 25%, and mesothelial cells of 10%. This was consistent with a transudative pleural effusion. The cytology and culture results of the fluid from thoracentesis were also negative. A transesophageal echocardiogram (TEE) showed thickening and elongation of the anterior leaflet of the mitral valve consistent with myxomatous degeneration, up to 1 cm in thickness at the margin of the anterior leaflet, prolapse of posterior leaflet into left atrium, aneurysm measuring 1 × 1.6 cm^2^ and perforation into left atrium and severe mitral regurgitation but no vegetations. The patient continued to have increased oxygen requirements and persistent hemoptysis and eventually underwent an emergent mitral valve repair. Perioperative TEE revealed hypertrophied right and left ventricles, normal right and left ventricular function with EF of 55%, mild anteroseptal wall hypokinesis, prolapse of the posterior mitral leaflet with a flail P3 segment, and severe mitral regurgitation with systolic flow reversal in right upper pulmonary vein (Figures 2(a) and 2(b)). A final diagnosis of alveolar hemorrhage secondary to severe acute mitral regurgitation from myxomatous degeneration of mitral valve was made. Notably, his hemoglobin had decreased from 14.5 gm/dL to 11.7 gm/dL. Postoperative TEE did not show any evidence of mitral valve regurgitation (Figure 2(c)). A repeat bronchoscopy 3 days after the mitral valve repair showed clearing of the alveolar hemorrhage (Figures 3(a), 3(b), and 3(c)). The patient rapidly recovered thereafter as was reflected in his CXR (Figure 4).

## 3. Discussion

Acute mitral regurgitation may occur as a result of four principal reasons: (1) a flail leaflet due to myxomatous disease, infective endocarditis, trauma, and so forth; (2) chordae tendineae rupture due to trauma, spontaneous rupture, infective endocarditis, and acute rheumatic fever; (3) papillary muscle rupture or displacement due to acute myocardial infarction or severe ischemia or trauma; (4) dysfunctional prosthetic valve due to degeneration, impaired closure, and paravalvular leak. Acute mitral regurgitation may present as cardiogenic shock and warrant urgent intervention [14]. There are several possible reasons for the development of DAH in acute mitral valve regurgitation. Hemodynamic changes in acute MR are more severe than in chronic MR. The degree of hemodynamic deterioration in acute MR depends upon the etiology and the degree of MR, but generally there is a lack of time for the left atrium and left ventricle to adapt. The normal left atrium is not compliant and the sudden and marked increase in left atrial volume in acute MR results in an abrupt elevation in pressure within the left atrium. This is immediately reflected back into the pulmonary circulation, often leading to pulmonary edema. TEE findings in patients with acute MR show that the regurgitant jet is directed at the right pulmonary venous system and that the velocity of flow and the pressure gradient is much greater at the orifice of the right pulmonary venous system compared with the left [15]. Transthoracic echocardiography may not provide accurate information about regurgitant volume and orifice area in acute MR when compared to chronic MR [16–18]. In some patients, DAH may present as a diffuse area of confluent ground glass opacity with sparing of the peripheral parenchyma, often referred to as “window frame” effect [11]. Chronic extravasation of blood from the capillaries due to conditions like chronic mitral stenosis (MS) can result in hemosiderosis and eventually pulmonary ossification [19, 20]. The case described here illustrates the varied manifestations of acute MR and the importance of considering acute MR as an etiology of DAH. Diagnostic procedures such as transesophageal echocardiography may help in an early diagnosis and assist in the decision to consider surgery as an early intervention.
